# Emerging New Crop Pests: Ecological Modelling and Analysis of the South American Potato Psyllid *Russelliana solanicola* (Hemiptera: Psylloidea) and Its Wild Relatives

**DOI:** 10.1371/journal.pone.0167764

**Published:** 2017-01-04

**Authors:** Mindy M. Syfert, Liliya Serbina, Daniel Burckhardt, Sandra Knapp, Diana M. Percy

**Affiliations:** 1 Natural History Museum, Department of Life Sciences, London, United Kingdom; 2 Naturhistorisches Museum, Basel, Switzerland; 3 Institut für Natur-, Landschafts- und Umweltschutz der Universität Basel, Basel, Switzerland; 4 Department of Botany, University of British Columbia, Vancouver, British Columbia, Canada; Agriculture and Agri-Food Canada, CANADA

## Abstract

Food security is threatened by newly emerging pests with increased invasive potential accelerated through globalization. The Neotropical jumping plant louse *Russelliana solanicola* Tuthill is currently a localized potato pest and probable vector of plant pathogens. It is an unusually polyphagous species and is widely distributed in and along the Andes. To date, introductions have been detected in eastern Argentina, southern Brazil and Uruguay. Species distribution models (SDMs) and trait comparisons based on contemporary and historical collections are used to estimate the potential spread of *R*. *solanicola* worldwide. We also extend our analyses to all described species in the genus *Russelliana* in order to assess the value of looking beyond pest species to predict pest spread. We investigate the extent to which data on geographical range and environmental niche can be effectively extracted from museum collections for comparative analyses of pest and non-pest species in *Russelliana*. Our results indicate that *R*. *solanicola* has potential for invasion in many parts of the world with suitable environmental conditions that currently have or are anticipated to increase potato cultivation. Large geographical ranges are characteristic of a morphological subgeneric taxon group that includes *R*. *solanicola*; this same group also has a larger environmental breadth than other groups within the genus. Ecological modelling using museum collections provides a useful tool for identifying emerging pests and developing integrated pest management programs.

## Introduction

Dispersal and distributional changes in crop pests (including vectored pathogens) poses a threat to both native and agricultural systems. Although much disease spread is human-mediated, latitudinal shifts in pest and pathogen distributions have been documented for a wide variety of groups [1–2]. These broad scale patterns suggest that climate change coupled with other environmental factors is having a significant impact on pest and pathogen distribution. Within these large datasets considerable variation has been observed for individual pest species [1], suggesting that detailed studies will reveal patterns that can be of importance for the prediction of outbreaks and control of disease in the future. Ecological modelling is increasingly applied to the investigation of threats from invasive species in non-native, agricultural and forestry systems [3–6]. Results from these studies on individual species predict distributional change and inform management subsequent to change, thereby mitigating or eliminating the negative impacts from invasions [7]. The Global Invasive Species Programme [8] advocates prevention measures and management plans be developed before invasive populations become established; this approach necessitates the study of species with invasive or pest potential. Agricultural and forestry pests are a particularly important focus for predictive analysis in order to assess areas of potential invasion, particularly given the possibility of outbreaks or distributional change mediated by human dispersal. Assessing climatic similarities between native and target regions can be used to predict species spread [7,9–12]. Species distribution models (SDMs) are a valuable approach for predicting invasion ecology by modelling climatic niches within native ranges and projecting the niche across a continental or global scale to identify regions with a higher likelihood of the species establishing. Biotic factors are also an important consideration [9,13], but such information is rarely available for taxa not yet on the threat radar.

Psyllids (Hemiptera: Psylloidea), also commonly called jumping plant-lice, comprise species that are important pests of a wide variety of crop plants. Psyllids damage plants both through their negative effects on plant growth when feeding and their potential to act as vectors for a variety of plant pathogens [14–17]. Psyllids are typically highly host specific, with immatures developing on a single host-plant, or a few closely related plant species [18–19]. In addition, host-plant switching is highly conserved, although psyllids are also known from a wide range of angiosperm families, suggesting host switching has occurred multiple times to unrelated plants during the evolution of the group [19–20]. The challenging nature of early identification and prediction of emerging psyllid pests is highlighted by the phylogenetically stochastic occurrence of known psyllid pests on a wide variety of plant taxa [19].

Potato (*Solanum tuberosum* L.; Solanaceae) is the third most important food crop worldwide [21] and provides calories and food security for populations in both the developed and developing countries. Potato is a member of the highly diverse genus *Solanum* that, with approximately 1500 species is one of a handful of plant genera with more than 1000 species [22]. Solanaceae and *Solanum* are most diverse in South America [23–24] but wild species occur on all continents except Antarctica. Other important food crops in Solanaceae include tomato (*Solanum lycopersicum* L.), tamarillo (*S*. *betaceum* Cav.), eggplant (*S*. *melongena* L.) and pepper (*Capsicum* spp.). Many pests and plant pathogens are specific to Solanaceae (e.g., the Colorado potato beetle, *Leptinotarsa decemlineata* Say; and the Irish potato famine pathogen, *Phytophthora infestans* (Mont.) de Bary) and host switching amongst crops and wild species may be common (e.g., [25]). Cultivar improvement in both potato and tomato for resistance to pests and pathogens has relied on knowledge of wild relatives, including their distribution and ecological characteristics.

Psyllids from eight genera are known to develop on Solanaceae [26–27]. The North American potato psyllid, *Bactericera cockerelli* Šulc (Triozidae) is one of the most destructive potato pests in the western hemisphere [28] and also occurs on several wild relatives of potato [29]. The potato disease ‘psyllid yellows’, first identified in the United States (see [30]) is vectored by *B*. *cockerelli*, and now affects other solanaceous crops such as tomatoes, pepper, eggplant and tamarillo in many parts of the world, causing severe economic losses [28,31–33]. *Bactericera cockerelli* has also recently been shown to be the vector of ‘zebra chip’, an emerging bacterial disease of potato [31,33]. *Bactericera cockerelli* is currently distributed and considered invasive [34] where it has been introduced by humans in areas of the Americas and New Zealand [35]. Another recently described psyllid species (*Acizzia solanicola* Kent & Taylor: Psyllidae) has been identified as a potentially serious commercial pest of eggplant in Australia [27,36]. It is one of several species of *Acizzia* associated with Solanaceae and has been recorded on both wild and cultivated species of *Solanum* [36]; to date there are no records of its associations with potatoes or tomatoes.

*Russelliana* (Psyllidae) is the only genus within the subfamily Aphalaroidinae that is associated with hosts in as many as eight plant families, suggesting a pattern of unusually wide and frequent host switching to unrelated plants [37–42]. *Russelliana solanicola* Tuthill was described from the solanaceous host plant *Datura* sp. in Peru, as well as from cultivated potatoes (*S*. *tuberosum*) where it caused feeding damage [41]. It has been reported as a pest on potato crops throughout Peru [43] and Chile [44] and is now considered a potentially serious emergent threat in South America [16,45–47]. It has also been identified as a possible future pest of tomato and pepper [48]. As with many recognized pests, *R*. *solanicola* exhibits a larger host range than fellow congeners; this pattern often differentiates non-pests from pests that have undergone a host range expansion [2,49].

*Russelliana solanicola* is an emerging and potentially global pest whose control may be better managed with early identification of the areas likely to be invaded. We model the global emergence potential of *R*. *solanicola*, and extend parts of the analysis to a comparison across the genus *Russelliana*. In order to better understand elements that differentiate pests from their non-pest relatives and investigate the emergence of pests within an evolutionary context, we explore the relevance of shared geographical, ecological and morphological characteristics of all *Russelliana* species. This approach has been useful in plant breeding where the characteristics of crop wild relatives (CWR) have been important for the identification of new tolerances to biotic/abiotic stresses and responses to climate change (e.g., [50–52]). Unlike studies of CWR in relation to crops, studies of insect pests are often limited to the target pest species, with little or no background information from related taxa and therefore there is little comparative data to inform our understanding of the evolutionary origins of pests.

Our objective is to harness museum specimen information and systematic and biogeographic knowledge of *Russelliana* to provide insights and predictions for an emerging pest. We test assumptions of pest-like ecology in *R*. *solanicola* and 18 described congeners with the aim of evaluating their potential for range expansion and invasion. We also investigate whether, given the patchiness of historical museum records, data on geographical range and environmental niche can be effectively extracted from museum collections for use in comparative analyses (e.g., between Solanaceae feeders and non-Solanaceae feeders as well as amongst morphological species groups within *Russelliana*). We evaluate species-environment relationships for *R*. *solanicola* and relatives with an ordination analysis of species co-occurrence and environmental variables.

## Materials and Methods

### Species and host background

The world psylloid fauna associated with Solanaceae is relatively species poor, with only 29 described species (from eight genera in four families) and at least 12 undescribed species [26–27,40,53–55]. However, a relatively large number of these are economically important pests and vectors of plant pathogens of crop plants, including potato, tomato and pepper. *Russelliana* has the largest number of Solanaceae feeding species; in addition to *R*. *solanicola*, four species (*R*. *capsici* Burckhardt, *R*. *disparilis* Tuthill, *R*. *fabianae* Burckhardt and *R*. *lycii* Tuthill) have Solanaceae hosts confirmed with immature material, and three species (*R*. *adunca* Burckhardt, *R*. *nigra* Burckhardt and *R*. *similis* Burckhardt) have no immature records or host data, but probably also develop on Solanaceae [37–39,40–42]. A further eight undescribed *Russelliana* species are most probably also associated with Solanaceae [40]. In our analyses, we have used only the 19 described species of *Russelliana* of which eight are or are likely to be Solanaceae feeding (see Table 1; [40,53]).

**Table 1:** Currently described *Russelliana* species with host-plant information and geographical range size.

Species	Number of localities	Host species	Morphological groups	Range area (km²)	Reference
*Russelliana adesmiae* Burckhardt	29	*Adesmia* spp. (Fabaceae)	3	34253	[37]
*Russelliana adunca* Burckhardt	3	?*Lycium* sp. (Solanaceae)	4	21475	[38]
*Russelliana bulbosa* Burckhardt	15	*Diostea juncea* (Gillies & Hook.) Miers (Verbenaceae)	clade A	607020	[38]
*Russelliana capsici* Burckhardt	6	*Capsicum annuum* L. (Solanaceae)	5	185448	[38,53]
*Russelliana chilensis* Burckhardt	3	?*Adesmia* sp. (Fabaceae)	3	1695	[38]
*Russelliana diosteae* Burckhardt	1	*Diostea juncea* (Gillies & Hook.) Miers (Verbenaceae)	clade A	78	[39]
*Russelliana disparilis* Tuthill	6	*Dunalia* sp. (Solanaceae)	4	754591	[38,42]
*Russelliana fabianae* Burckhardt	26	*Fabiana imbricata* Ruiz & Pav. (Solanaceae)	1	129980	[38]
*Russelliana intermedia* Burckhardt	2	?Asteraceae	6	157	[38]
*Russelliana lycii* Tuthill	2	*Lycium salsum* Ruiz & Pav. (Solanaceae)	5	157	[41] as *Arepuna lycii*, [38]
*Russelliana maculata* Burckhardt	2	?*Adesmia* sp. (Fabaceae)	3	157	[38]
*Russelliana marionae* Burckhardt	1	*Mulguraea scoparia* (Gillies & Hook.) N.O'Leary & P.Peralta (Verbenaceae)	clade A	78	[39]
*Russelliana nigra* Burckhardt	2	?Solanaceae	4	157	[38]
*Russelliana punctulata* Burckhardt	1	?*Adesmia* sp. (Fabaceae)	3	78	[38]
*Russelliana sebastiani* Burckhardt	5	*Diostea juncea* (Gillies & Hook.) Miers (Verbenaceae)	clade A	1333	[39]
*Russelliana similis* Burckhardt	2	?Solanaceae	4	157	[38]
*Russelliana solanicola* Tuthill	91	*Alternanthera ficoidea* (L.) Sm. (Amaranthaceae), *Baccharis* spp. (Asteraceae), *Helenium aromaticum* (Hook.) L.H.Bailey (Asteraceae), *Parthenium hysterophorus* L. (Asteraceae), *Xanthium spinosum* L. (Asteraceae), *Escallonia rosea* Griseb. (Escalloniaceae), *Brugmansia arborea* (L.) Steud. (Solanaceae), *Datura* sp. (Solanaceae), *Solanum tuberosum* L. (Solanaceae)	4	2496773 6277176*	[38,41,46]
*Russelliana theresae* Burckhardt	6	*Mulguraea scoparia* (Gillies & Hook.) N.O'Leary & P.Peralta (Verbenaceae)	clade A	52	[39]
*Russelliana vinculipennis* Burckhardt	5	?*Adesmia* sp. (Fabaceae)	3	140553	[38]

Morphological groups correspond to the preliminary clades identified by [40]: potential hosts of eight species are marked with a question mark (?). The range area of *R*. *solanicola* that includes localities where it is likely introduced is marked with an asterisk (*)

Phylogenetic work based on morphology indicates there are two primary species groups within the genus *Russelliana*. The first is relatively well defined (“group 1”) and sister to the rest, while the second group comprises several subgroups (“group 3”, “group 4” of which *R*. *solanicola* is a member, “group 5”, “group 6”, and “clade A”, according to [38–40]. Morphological group membership of currently described *Russelliana* species is indicated in Table 1.

### Occurrence data

We databased all specimen label data for *Russelliana* from the Natural History Museum, London, United Kingdom (BMNH), the Muséum d'histoire naturelle, Geneva, Switzerland (MHNG), the Naturhistorisches Museum, Basel, Switzerland (NHMB), and the California Academy of Sciences (CAS); 24 records with geographic coordinates were added from literature reports. Where geographic coordinates were not given on labels, records were georeferenced based on locality descriptions. The accuracy of georeferencing varied from within 500 m to over 100 km with a median error of less than 8 km. Our dataset includes a total of 208 records (i.e., unique localities per species) of *Russelliana* representing 2564 specimens for all 19 currently described species (Table 1). Of the 76 host-plants listed in these records, 53 are unconfirmed due to the absence of immature records or host data (probable hosts for these taxa listed in Table 1 are based on [40]). All records represent native ranges for *Russelliana* species, with the exception of seven localities where *R*. *solanicola* occurs in eastern South America and has been assessed as non-native (see [46]). However, the exact provenance of the specimen from one non-native locality, in Uruguay, is unknown and for this reason we exclude it from further analysis.

### Environmental data

We selected environmental variables likely to affect psyllid biology (following [56]) in order to build species distribution models (SDMs), investigate the environmental space of *Russelliana* species, and explore the relationship between *Russelliana* species co-occurrence and environmental variables. To build the SDMs for *R*. *solanicola*, we used: 1) mean annual precipitation, mean annual temperature, precipitation seasonality, and temperature seasonality from the WorldClim database version 1.4 (http://www.worldclim.org; [57]) (http://www.worldclim.org; Hijmans et al., 2005); and 2) mean monthly Enhanced Vegetation Index (EVI) derived from Moderate Resolution Imaging Spectrometer (MODIS) satellite data and geological ages based on the surface geology from WorldGrids (http://www.worldgrids.org; accessed October 1, 2015). For the additional environmental space and species-environment relationships analyses, we obtained mean annual potential evapotranspiration (PET) and mean annual actual evapotranspiration (AET) from CGIAR consortium for spatial information (http://www.cgiar-csi.org/; [58]) as well as calculated annual water deficit (PET-AET; [59]). All of the above variables had a 30 arc second (~1 km at the Equator) spatial resolution.

### Species distribution modelling

A species distribution model (SDM) was built for the pest species *R*. *solanicola* with georeferenced specimen records as occurrence data. A sampling bias correction was applied to the occurrence data by using a method advocated by Fourcade et al. [60] in which the occurrence data are systematically selected across a regularly distributed geographical space to reduce the spatial aggregation of records. We systematically subsampled the records using a distance of 15 km for a total of 57 *R*. *solanicola* occurrences. MaxEnt (version 3.3.3k; [61]) was selected to build the SDM because it is an effective modelling approach when utilizing presence-only data from museum collections [62]. Our models were trained with *R*. *solanicola* occurrences within its native range and 15,000 background points constrained to the geographical extent of the native points (i.e., western South America). We adopted the default regularisation parameters but restricted MaxEnt to using only linear and quadratic functional forms, which constrains models to produce relatively simple models that do not over-fit to the training data [63–64]. The SDM was built using a 5-fold cross–validation approach to assess model predictive accuracy. This approach uses 80% of the data to train the model and reserves 20% for model evaluation; this process was repeated until each reserved set was used to evaluate the models [65]. SDM performance was evaluated by using the area under the curve (AUC) in a receiver operating characteristic (ROC) plot; an AUC value of 1.0 indicates perfect discrimination ability and a value of 0.5 or less indicates a prediction no better than random. Binary maps of predicted presence–absence were created for each replicate model using a maximum sensitivity and specificity threshold [66–67]. The final SDM output was an ensemble of the cross–validation models; a grid cell was considered a presence when 3 or more models predicted a presence. We also evaluated the predictive performance of the SDM with six introduced localities of *R*. *solanicola*. A jack-knife procedure (leave one out) was used to evaluate variable importance in the models. We compared our *R*. *solanicola* SDM to a model of suitable climates for the cultivated potato adapted from previous studies [68–69].

### Measuring geographical range and data analyses

Geographical range was estimated by applying a minimum convex polygon (MCP) around known species locations. This aspect of the geographical range is the area that lies within the outermost limits of the known locations. We have reasonable confidence that most of the species are well sampled and the known locations of the species represent its true geographical spread. Eight species (*R*. *diosteae* Burckhardt, *R*. *intermedia* Burckhardt, *R*. *lycii*, *R*. *maculata* Burckhardt, *R*. *marionae* Burckhardt, *R*. *nigra*, *R*. *punctulata* Burckhardt, and *R*. *similis*) have fewer than three known localities and a MCP could not be calculated (see Table 1); for these taxa we created 5 km buffers around each locality. A Principal Component Analysis (PCA) was performed to distinguish possible environmental gaps or clusters between all taxa considered Solanaceae feeding (eight) and non-Solanaceae feeding species (eleven) of *Russelliana*. In addition, the PCA was used to identify environmental patterns amongst the morphological groups within the genus (defined based on the preliminary phylogenetic study of *Russelliana*; [40], see above).

We investigated *Russelliana* species co-occurring at the sampling sites and whether these assemblages were associated with environmental variables. Non-metric multidimensional scaling (NMDS) is considered a robust unconstrained ordination method in community ecology [70] and iteratively ranks datasets according to their pairwise dissimilarity and is well suited to non-normal data [71]. NMDS analyses use a matrix of dissimilarities between pairs of sampling sites based on compositional data. The compositional data in these analyses are typically plot-based, here we analysed *Russelliana* species co-occurrence by standardizing presence-only data, grouping the data into sampling units of 25 km grid cells and creating a presence–absence matrix from all locations that had a least one species of *Russelliana* present. For the NMDS ordination, the binomial distance measure was used to generate the dissimilarity matrix [72]. Optimal NMDS configurations were determined using 150 random starts, and ordinations with the lowest stress values were used. Environmental variables selected for this study were incorporated into the analysis through the use of bi-plot ordinations in which variables were plotted as vector fits against co-occurrence assemblage ordinations. Permutation tests (999) were used to determine the significance of vector fits with ordination axes, and significant (P < 0.001) variables were included in the resulting bi-plots. The sample positions were evaluated within the ordination by including convex hulls to investigate the potential existence and distinctness of spatially or environmentally determined Solanaceae feeding and non-Solanaceae feeding assemblages.

### Morphological trait data analysis

In order to investigate the significance of morphological characters thought to be ecologically relevant for psyllid biology between Solanaceae feeders and non-Solanaceae feeders we extracted trait data from [46] and measured the same traits from newly acquired specimens of *R*. *capsici* and *R*. *lycii*. We measured a total of 186 specimens. We investigated the relationship between range size and median morphological characters for each of the 19 *Russelliana* species. We focused on six potentially ecologically relevant morphological traits: genal processes length, head width, antennal length, forewing length and forewing width (potential indicators of active or passive dispersal modes), and forewing length/head width ratio (an indicator of body mass).

All data analyses were performed using R 3.0.2 [73]. We implemented the NMDS ordination using the vegan package for R [74]. Vector fitting of variables within ordinations were performed using the envfit function within the vegan package. All map outputs were created in ESRI ArcGIS 10 [75].

## Results

### Species distribution model (SDM)

**Fig 1:**
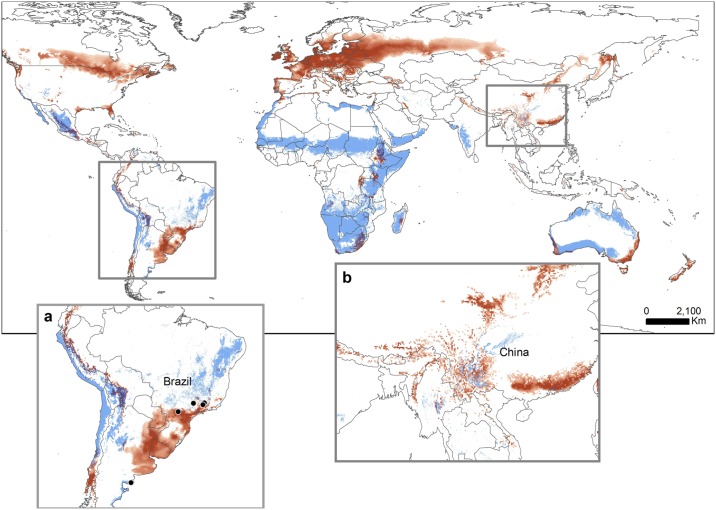
Predicted suitable habitat for *R*. *solanicola*. Predicted suitable habitat for *R*. *solanicola* (blue, predicted as presence/absence), using the MaxEnt species distribution modelling (SDM) approach overlayed with predicted suitable habitat for the cultivated potato (*S*. *tuberosum*; (red, darker shades represent higher suitability)), using data from Schafleitner et al. [69]; areas of overlap in regions such as central Mexico and eastern South Africa are shown in dark grey; a) *R*. *solanicola* and potato geographical overlap in the Andes and parts of eastern South America, black points represent recent confirmed introductions of *R*. *solanicola*; b) *R*. *solanicola* and potato geographical overlap in southern China.

Predictive performance of the *R*. *solanicola* SDMs was good with all five replicate models having an AUC of 0.77 and above (test AUC range: 0.77–0.88). The jack–knife procedure suggested mean EVI (i.e., sensitivity to mean vegetation cover) was the most important variable in the SDMs, followed by geological age (a potential indicator of soil composition) and mean annual temperature (S1 Table). *Russelliana solanicola* occurrences had a quadratic relationship with EVI, with values ranging from 0 (i.e., bare rocks) to 100,000 (i.e., dense forest canopy), however, optimal probability of occurrence ranged from 800–2,500; the dominant geological age was Triassic/Jurassic (~250–200 million years ago) and optimal mean annual temperature was between 15–22°C. The final SDM based on an ensemble of the presence–absence models represents well the known distribution of the species, including the region in Brazil where the species is considered to be introduced (Fig 1); of the six introduced sites, five (83%) are accurately predicted as a presence. Globally, regions that grow potatoes as a crop and are climatically suitable for *R*. *solanicola* include western South America, Mexico, southern and eastern Africa, central and south-eastern Asia, and southern Australia (Fig 1).

### Geographical ranges

**Fig 2:**
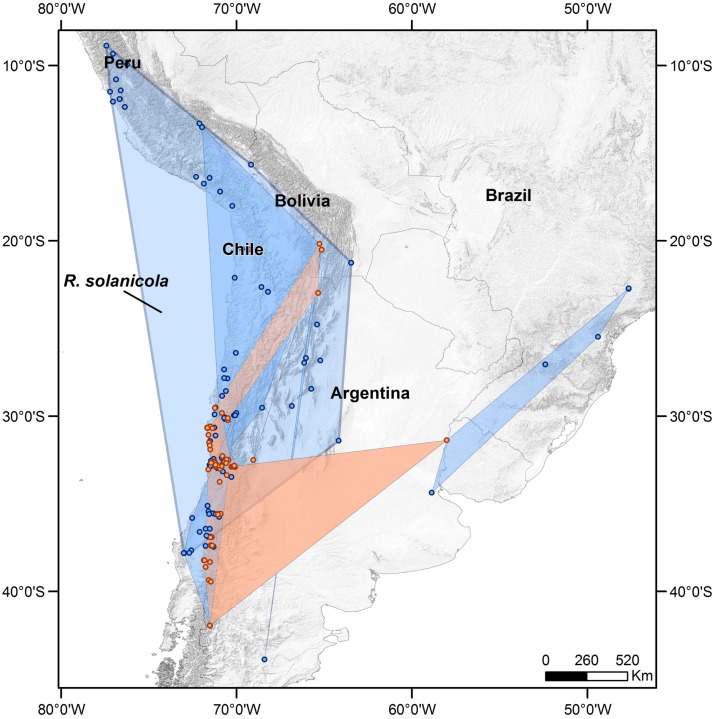
Native ranges of *Russelliana* species. Native ranges of eight Solanaceae feeding species (blue points and polygons) and eleven non-Solanaceae feeding species (orange points and polygons) of *Russelliana* in western and eastern South America as derived from minimum convex polygon (MCP). The native range for the pest species *R*. *solanicola* is labeled.

*Russelliana* species occur throughout South America. The geographical ranges (as measured by the minimum convex polygon) of the described *Russelliana* species are concentrated across southern South America with many ranges that follow the Andes mountains along the border between Chile and Argentina (Fig 2). The range of *R*. *capsici* is exclusively in eastern South America, occurring primarily in Brazil with one occurrence in eastern Argentina. The potato pest, *R*. *solanicola*, has the largest native geographical range (Table 1); inclusion of introduced localities from Brazil and eastern Argentina increase this range size by four times. The native range alone is more than double the size of the next largest range (*R*. *disparilis*, which may have an even more restricted distribution as the records from eastern South America are based only on female specimens). The largest four native ranges of the 19 *Russelliana* species are from three morphologically defined groups (Table 1), and three (*R*. *capsici*, *R*. *disparilis* and *R*. *solanicola*) of these four species are associated with Solanaceae. Further work is needed to confirm phylogenetic independence of shared geographical ranges, dispersal ability, and host-plants in *Russelliana*, but our preliminary data emphasize the value of looking at pest biology in the broader context of related taxa. The present morphological groups suggest there are as many as three independent colonizations of Solanaceae: *R*. *capsici* and *lycii* (“group 5”), *R*. *fabianae* (“group 1”) and *R*. *solanicola* and related species (“group 4”) (Table 1; [40]).

### Environmental data analysis

**Fig 3:**
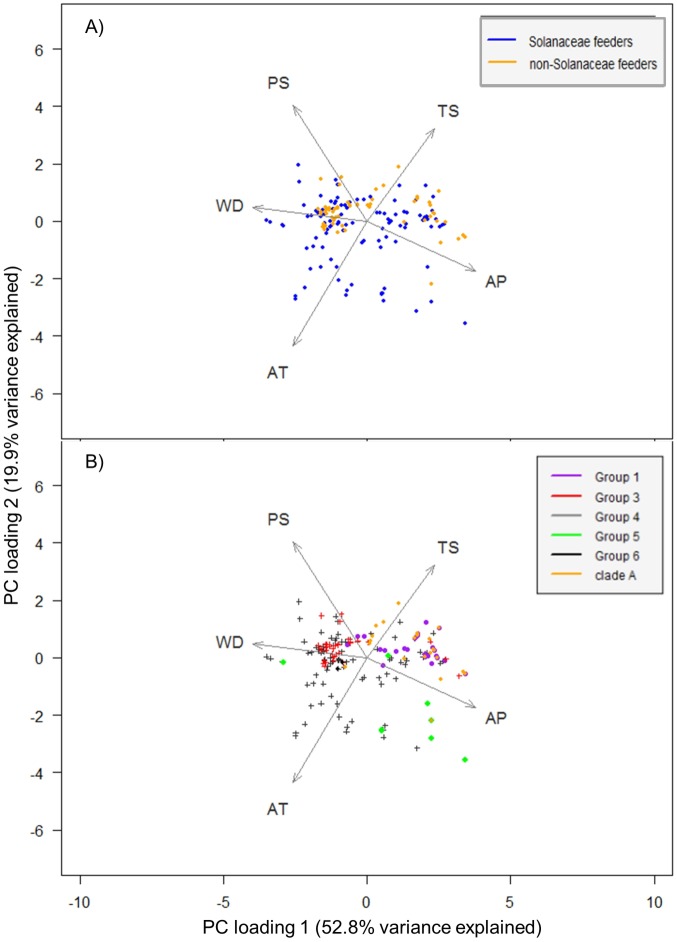
Principal component analysis (PCA). Principal component analysis (PCA) of Solanaceae feeding species and non-Solanaceae feeding species: (A) on axes 1 and 2; (B) PCA of morphological groups (see Table 1 for *Russelliana* group membership) on axes 1 and 2. Environmental variables codes: AP = annual precipitation, AT = annual temperature, WD = annual water deficit, PS = precipitation seasonality, TS = temperature seasonality.

The PCA reveals a pattern of Solanaceae feeding species distributed across a somewhat larger environmental space than non-Solanaceae feeding species (Fig 3A). Morphological groups identified within *Russelliana* tend to cluster more within environmental space, except group 4, which includes *R*. *solanicola* (Fig 3B). It is possible, however, that the larger number of *R*. *solanicola* records to some extent influences this lack of clustering.

### Co-occurrence assemblages

**Fig 4:**
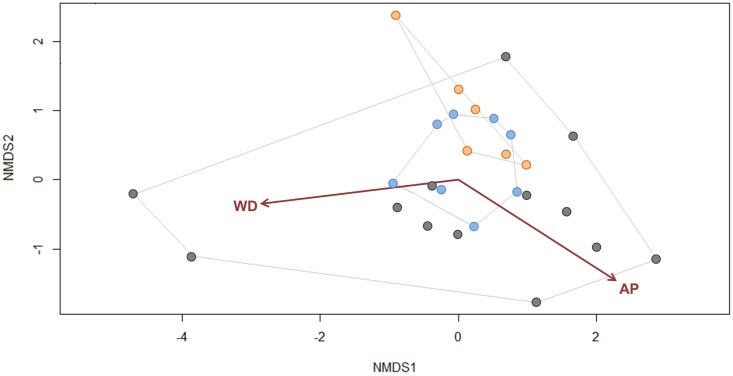
Non-metric Multidimensional Scaling (NMDS) plot. Non-metric Multidimensional Scaling (NMDS) plot illustrating co-occurrence dissimilarity of 19 *Russelliana* species between sample sites (derived from 25 km grid). Group boundaries for three *Russelliana* co-occurrence assemblages: Solanaceae feeding (blue points), non-Solanaceae feeding (orange points), and an assemblage with Solanaceae feeding species as well as non-Solanaceae feeding species (dark grey points) were drawn using ordihull function in the R package vegan [74]. Environmental variables significantly correlated with NMDS ordination are shown: water deficit (WD) and mean annual precipitation (AP).

Most points on the NMDS ordination are dispersed and indicate that co-occurrence assemblages vary considerably among sites (Fig 4; *k* = 2, stress = 0.05), however, psyllid co-occurrence assemblages overlap in ordination space when we investigated discrete groupings (i.e., sites with only Solanaceae feeding species, sites with only non-Solanaceae feeding species, and sites that includes Solanaceae feeding species and non-Solanaceae feeding species). Two environmental variables were significantly correlated with *Russelliana* co-occurrence assemblages: mean annual precipitation and annual water deficit (Fig 4). Although these relationships are statistically significant, they were weak with low *R*^*2*^ (annual water deficit R^*2*^ = 0.19, *p-value* < *0*.*001*; mean annual precipitation *R*^*2*^ = 0.17, *p-value* < *0*.*001)*. This indicates that water deficit and annual precipitation contribute to explaining a small amount of variation in *Russelliana* co-occurrence assemblages but not enough to generate predictions.

### Morphological trait analysis

Evaluations of character measurements across species of *Russelliana* indicate that *R*. *solanicola* tends to have characters with median values that are distinct from those of other species, although there is also considerable overlap (S1a and S1b Fig). Taylor and Kent [27] found morphological differences (presence/absence of genal processes, antennal length) between Solanaceae feeding versus non-Solanaceae feeding species in the psyllid genus *Acizzia*; we did not find support for similar differences in *Russelliana* in either these two characters or in head width, forewing length, forewing width and forewing length/head width ratio. The relationships between all morphological characters and geographical range size were very weak (S2 Fig). Our data do not support morphological characters as indicators of geographical or environmental patterns in *Russelliana*; however the scale of our character measurements may be too coarse to be informative (i.e., psyllids are extremely small, ~2–3 mm) or the characters selected are not optimally informative for the variables analysed.

## Discussion

Our model for climatic suitability of the potentially invasive South American potato psyllid identifies several regions suitable for *R*. *solanicola* colonization that are outside its native range, suggesting a greater risk of invasion and establishment in these regions (Fig 1). The best predictors for *R*. *solanicola* occurrence are mean EVI, geology and mean annual temperature (S1 Table). *Russelliana solanicola* occurrences were best predicted when the vegetation index (i.e., EVI) ranged between sparse vegetation to open canopy as well as areas with moderate temperatures and moderately old surficial geology.

*Russelliana solanicola* is already introduced in eastern Argentina, southern Brazil and Uruguay, and is thought to be in the incipient stages of invasion in these regions, which stresses the importance of early detection. Established understanding of human-mediated spread resulting in the global spread of the North American potato psyllid (*B*. *cockerelli*) [48] suggests that, given time, *R*. *solanicola* could have similar detrimental impacts on potato cultivation in many new areas, such as those recently recognized as highly suitable for potato cultivation [68] (Fig 1).

Potato cultivation is expanding in many parts of the world previously not considered suitable, such as southwestern China. China is the world’s leading producer of potatoes [76] and the Ministry of Agriculture has recently (early 2015, see http://www.chinadaily.com.cn/china/2015-01/09/content_19278303.htm) indicated a desire for increase in cultivation and consumption of potatoes in the country. New varieties developed specifically for use in both low and high elevation areas of southwestern China [77] are contributing to a dramatic increase in area under potato cultivation in both winter and summer growing seasons. Although new varieties are highly resistant to a number of common pathogenic diseases [77] producers may need to consider the risks and prevention of *R*. *solanicola* invasion and concomitant disease risk; our models indicate these regions as climatically suitable for, but not yet occupied by, *R*. *solanicola*. Areas of southern Africa are also potential regions for this combination of increased potato cultivation [69] and potential *R*. *solanicola* invasion (see Fig 1). Understanding the threat level for this emerging potato pest is particularly important given its role as a potential vector of plant pathogens [16,43,45,47].

Our model of climate matching between native and non-native areas is an initial step in evaluating the risk of invasion [78]. Our SDMs, built with empirical data, model the realized niche [7]. The realized niche is shaped by both abiotic factors and biotic interactions that modulate species distributions; due to the nature of our dataset we only included abiotic variables in our SDM. Potentially important biotic interactions were not considered due to the current lack of knowledge of these parameters for most species of psyllid. These include factors such as competition with local species in the invaded region, the presence/absence of natural enemies (predators, diseases, and parasitoids), and/or population recruitment and establishment. Acquiring knowledge on psyllid biology is hampered by the fact that these are small insects often overlooked in general collecting for biodiversity assessment. Focused efforts to close knowledge gaps between native/non-native biologies/populations, as well as between target/pest and non-target/wild relatives are needed to improve predictive modelling. Such efforts will support acquisition of critical background biological data enabling predictive modelling that takes into account biological factors. Our model is thus only a relatively coarse approximation of the niche projected outside of the native range, and it is possible that areas with low predicted suitability could also sustain *R*. *solanicola* populations, especially if those areas were currently unsuited or uninvaded by other Solanaceae feeders or potential competitors.

Our comparison of *R*. *solanicola* and other *Russelliana* species indicates some shared similarities in geographical and environmental characteristics, and among morphological groups. Although we did not find morphological traits specifically associated with Solanaceae feeding species (contrary to [27]), we did find support for sites with Solanaceae feeding species having larger environmental breadth than sites with non-Solanaceae feeding species. These findings suggest that future studies on shared environmental tolerances among assemblages of taxa may be useful for framing questions on the relevance of environmental conditions for species distributions. The fairly subtle differences in environmental tolerances found here may yet be important in determining the establishment and survival of different psyllid groups. In addition, there is a general overlap of wild potato species with *R*. *solanicola* and its relatives (S3 Fig) in southern South America. Further assessment of range comparisons using combined data resources for *Russelliana* and other psyllid species, with data for wild relatives of potato, could be useful in future exploration of potato feeding insects and their propensity to switch from wild to cultivated hosts.

It has long been recognized that natural history museum collections provide important historical records [79–86] for understanding distributions of species in relation to a variety of environmental factors. Accurate locality data coupled with broad geographical coverage in space can provide powerful baseline data for modelling approaches [87]. Our study utilizes a combination of natural history museum collections and expert taxon knowledge to assess potentially important ecological characteristics of putative pests. We have considered the biogeographical and environmental components but these are only a piece of what is a complex puzzle. In order to provide more accurate predictions, we would need more detailed site-specific biological information, such as plant chemical analyses, physiological tolerance experiments, and more detailed ecological information about *Russelliana* species. Unfortunately, these important factors are usually only investigated when the species in question is already recognized as a serious pest. Predictions of future pest invasions or outbreaks in new areas rely on patchy information, and therefore museum specimens, even if few, can provide baseline information useful for early detection and management, analogous to their use for emerging infectious diseases [88]. As our food supply becomes more homogenous [89] and major crops such as potato are grown in areas whose suitability changes as climate shifts [69,90], identification and detection of potential and emerging pests becomes ever more important for food security worldwide. The digitisation and open availability of museum collections (e.g., papers in [91]) will be an important part of the solution to this societal problem.

## Supporting Information

S1 Table Environmental variables. Average environmental variable importance of *Russelliana solanicola* SDMs (5 replicate models).
(PDF)

S2 Table Georeferenced *Russelliana* specimens. Specimen records in museum collections are as follows: Natural History Museum, London (BMNH), California Academy of Sciences (CAS), Museum d'Histoire Naturelle, Geneva (MHNG), Naturhistorisches Museum, Basel (NHMB) [38,41,92].
(PDF)

S1 Fig **S1a Fig. Morphological traits.** Boxplots of morphological traits for *Russelliana* species; Solanaceae feeding species (blue) and non-Solanaceae feeding species (orange). **S1b Fig. Morphological traits.** Boxplots of morphological traits for *Russelliana* species; Solanaceae feeding species (blue) and non-Solanaceae feeding species (orange).
(PDF)

S2 Fig Linear regressions morphological traits. Linear regressions of median values of morphological traits and geographical ranges for 19 *Russelliana* species.
(PDF)

S3 Fig Co-occurrence of *Russelliana* and potato wild relatives. Known geographical localities of 19 *Russelliana* species from Argentina, Bolivia, Brazil, Chile, Peru and Uruguay, overlapping with point data (grey) of potato crop wild relatives (CWR) available from Solanaceae Source (http://solanaceaesource.org/). Blue points represent Solanaceae feeding species and orange points represent non-Solanaceae feeding species.
(PDF)
