# Supplementary material for: Emerging New Crop Pests: Ecological Modelling and Analysis of the South American Potato Psyllid Russelliana solanicola (Hemiptera: Psylloidea) and Its Wild Relatives
Source: PLoS One. 2017 Jan 4;12(1):e0167764. doi: 10.1371/journal.pone.0167764 (PMC5214844; doi:10.1371/journal.pone.0167764)
Supplement: S1 Table — Average environmental variable importance of Russelliana solanicola SDMs (5 replicate models). (PDF) [file pone.0167764.s001.pdf]

| Variable | Percent contribution |
|---|---|
| Enhance Vegetation Index | 51 |
| Geology | 18.5 |
| Mean annual temperature | 16.2 |
| Temperature seasonality | 7.5 |
| Mean annual precipitation | 4 |
| Precipitation seasonality | 2.8 |

**S1 Table. Environmental variables.** Average environmental variable importance of *Russelliana solanicola* SDMs (5 replicate models).
