# Supplementary material for: Emerging New Crop Pests: Ecological Modelling and Analysis of the South American Potato Psyllid Russelliana solanicola (Hemiptera: Psylloidea) and Its Wild Relatives
Source: PLoS One. 2017 Jan 4;12(1):e0167764. doi: 10.1371/journal.pone.0167764 (PMC5214844; doi:10.1371/journal.pone.0167764)
Supplement: S2 Table — Specimen records in museum collections are as follows: Natural History Museum, London (BMNH), California Academy of Sciences (CAS), Museum d'Histoire Naturelle, Geneva (MHNG), Naturhistorisches Museum, Basel (NHMB) [38,41,92]. (PDF) [file pone.0167764.s002.pdf]

Species	Country	Lat.	Long.	Date	Collector	Museum
adesmiae	Chile	-33.3775	-70.5624	9.i.1985	D Hollis	BMNH
adesmiae	Chile	-33.3775	-70.5624	9.i.1985	D Hollis	BMNH
adesmiae	Chile	-30.5989	-71.5065	17.ii.1985	D Hollis	BMNH
adesmiae	Chile	-30.5989	-71.5065	17.ii.1985	D Hollis	BMNH
adesmiae	Chile	-31.5088	-71.5659	22.v.1986	J Solervicens	MHNG
adesmiae	Chile	-31.5088	-71.5659	20.viii.1985	J Solervicens	MHNG
adesmiae	Chile	-31.0723	-71.6062	20.viii.1985	M Elgueta	MHNG
adesmiae	Chile	-31.9018	-71.4969	2.xii.1993	D Burckhardt	MHNG
adesmiae	Chile	-31.0723	-71.6062	23.i.1986	M Elgueta	MHNG
adesmiae	Chile	-29.8333	-70.8333	4.xii.1993	D Burckhardt	NHMB
adesmiae	Chile	-29.8333	-70.8333	4.xii.1993	D Burckhardt	MHNG
adesmiae	Chile	-30.6667	-71.6667	18.ii.1985	D Hollis	BMNH
adesmiae	Chile	-30.7024	-71.3761	17.ii.1985	D Hollis	BMNH
adesmiae	Chile	-30.7024	-71.3761	17.ii.1985	D Hollis	BMNH
adesmiae	Chile	-30.6808	-71.6591	7-9.xii.1990	D Agosti & D Burckhardt	MHNG
adesmiae	Chile	-30.6667	-71.6667	14-16.xii.1992	D Burckhardt	NHMB
adesmiae	Chile	-30.6657	-71.6808	15.xii.1992	D Burckhardt	NHMB
adesmiae	Chile	-30.6667	-71.6667	16.xii.1992	D Burckhardt	NHMB
adesmiae	Chile	-30.6667	-71.6667	15-17.xii.1992	D Burckhardt	NHMB
adesmiae	Chile	-32.9000	-70.3000	31.xii.1993	D Burckhardt	MHNG
adesmiae	Chile	-33.7549	-70.9635	4.v.1993	D Burckhardt	MHNG
adesmiae	Chile	-33.0167	-70.9333	28.xii.1993	D Burckhardt	MHNG
adesmiae	Chile	-31.6756	-71.5268	23.i.1986	M Elgueta	BMNH
adesmiae	Chile	-32.2823	-70.8124	21-26.xii.1998	D Burckhardt	NHMB
adesmiae	Chile	-32.6067	-71.2400	23.ii.2009	D Burckhardt	NHMB
adesmiae	Chile	-32.5969	-71.2440	22.xii.1995	D Burckhardt	MHNG
adesmiae	Chile	-32.6667	-71.2167	22.xi.1992	D Burckhardt	NHMB
adesmiae	Chile	-32.8667	-71.2167	1.i.1994	D Burckhardt	MHNG
adesmiae	Chile	-32.8896	-71.1801	30.xii.1998	D Burckhardt	NHMB
adesmiae	Chile	-32.8896	-71.1801	30.xii.1998	D Burckhardt	NHMB
adesmiae	Chile	-32.8667	-70.8500	28.xii.1993	D Burckhardt	MHNG
adesmiae	Chile	-32.8354	-71.0720	24.xii.1995	D Burckhardt	MHNG
adesmiae	Chile	-32.5091	-70.4566	1.i.1999	D Burckhardt	NHMB
adesmiae	Chile	-32.7868	-71.1979	26.xii.1995	D Burckhardt	MHNG
adesmiae	Chile	-32.6167	-70.7000	26.xii.1993	D Burckhardt	MHNG
adesmiae	Chile	-32.4867	-70.5650	6.iii.2009	D Burckhardt	NHMB
adesmiae	Chile	-32.6844	-70.5946	21.i.1997	D Burckhardt	NHMB
adesmiae	Chile	-32.6667	-71.4500	22.xi.1992	D Burckhardt	NHMB
adesmiae	Chile	-32.6667	-71.4500	22.xi.1992	D Burckhardt	NHMB
adunca	Argentina	-43.8858	-68.4115	3.x.1987	LE Peña	MHNG
adunca	Bolivia	-20.1739	-65.2731	26–27.xii.1984	LE Peña	MHNG
adunca	Bolivia	-20.5107	-65.1485	26–28.xii.1984	LE Peña	MHNG
bulbosa	Chile	-39.3616	-71.5890	26.i.1985	D Hollis	BMNH
bulbosa	Chile	-37.3941	-71.4072	19.i.1996	D Burckhardt	MHNG
bulbosa	Chile	-37.3766	-71.4732	22.i.1996	D Burckhardt	MHNG
bulbosa	Argentina	-31.3719	-58.0156	10.iii.2010	JP Bouvet	NHMB
bulbosa	Chile	-39.4450	-71.4678	26.i.1985	D Hollis	BMNH
bulbosa	Chile	-38.2282	-71.8626	23.i.1996	D Burckhardt	MHNG
bulbosa	Chile	-38.2252	-71.8233	23-27.i.1996	D Burckhardt	MHNG
bulbosa	Argentina	-41.9549	-71.5325	1.iv.1961	G Topal	MHNG
bulbosa	Argentina	-41.9549	-71.5325	1.iv.1961	G Topal	MHNG
bulbosa	Chile	-35.5833	-71.0667	4-5.i.1994	D Burckhardt	MHNG

bulbosa	Chile	-35.5715	-70.9158	14.i.1996	D Burckhardt	MHNG
bulbosa	Chile	-35.6275	-71.0723	13.i.1996	D Burckhardt	MHNG
bulbosa	Chile	-35.6275	-71.0723	13.i.1996	D Burckhardt	NHMB
bulbosa	Chile	-35.6153	-71.0557	12.i.1996	D Burckhardt	MHNG
bulbosa	Chile	-36.9091	-71.4188	15.i.1985	D Hollis	BMNH
bulbosa	Chile	-33.0521	-71.6113			Burckhardt, 1987 [38]
bulbosa	Chile	-37.4228	-71.3645	19.i.1985	D Hollis	BMNH
bulbosa	Chile	-36.9091	-71.4188	15.i.1985	D Hollis	BMNH
capsici	Argentina	-34.3698	-58.8701	i.1943	JB Daguerre	MHNG
capsici	Brazil	-25.4750	-49.3919	27-30.xi.2012	D Burckhardt & DL Queiroz	NHMB
capsici	Argentina	-31.3719	-58.0156	10.iii.2010	JP Bouvet	MHNG
capsici	Brazil	-22.7343	-47.6481	1.viii.1969-1.ix.1969	Max de Meneres	BMNH
capsici	Brazil	-27.0500	-52.4000	26.viii.1943	F Plaumann	BMNH
capsici	Brazil	-22.7070	-47.6433	viii-ix.1969	HD Catling	MHNG
capsici	Brazil	-22.7070	-47.6433	viii-ix.1969	H Catling	BMNH
chilensis	Chile	-38.6060	-71.7594	30.i.1996	D Burckhardt	MHNG
chilensis	Chile	-38.3360	-71.5115	8.iii.1989	T Cekalovic	MHNG
chilensis	Chile	-36.9202	-71.4931	12-13.xii.1983	LE Peña	MHNG
chilensis	Chile	-36.9202	-71.4931	12–13.xii.1983	LE Peña	MHNG
diosteae	Chile	-35.5715	-70.9158	14.i.1996	D Burckhardt	NHMB
disparilis	Bolivia	-20.1739	-65.2731	26–27.xii.1984	LE Peña	MHNG
disparilis	Bolivia	-20.5107	-65.1485	26–28.xii.1984	LE Peña	MHNG
disparilis	Bolivia	-20.5107	-65.1485	26–28.xii.1984	LE Peña	MHNG
disparilis	Peru	-13.5155	-71.9663	13.vi. 1959	D L Tuthill	Burckhardt, 1987 [38]
disparilis	Chile	-30.1048	-70.7105	18.xii.1995	D Burckhardt	MHNG
disparilis	Argentina	-29.5255	-68.5396	1/3.xii.1983	LE Peña	MHNG
disparilis	Chile	-32.3842	-70.8518	21.xii.1998	D Burckhardt	NHMB
disparilis	Bolivia	-20.1739	-65.2731	26/27.xii.1984	LE Peña	NHMB
fabianae	Chile	-35.4342	-71.6065	14.ii.1985	D Hollis	BMNH
fabianae	Chile	-37.4120	-71.7709	21.xi.1981	RT Schuh & NI Platnick	NHMB
fabianae	Chile	-37.3766	-71.4732	22.i.1996	D Burckhardt	NHMB
fabianae	Chile	-37.3941	-71.4072	19.i.1996	D Burckhardt	MHNG
fabianae	Chile	-37.3968	-71.4262	21.i.1996	D Burckhardt	NHMB
fabianae	Chile	-37.3968	-71.4262	21.i.1996	D Burckhardt	MHNG
fabianae	Chile	-37.4603	-71.3103	21.i.1996	D Burckhardt	NHMB
fabianae	Chile	-35.8167	-72.5333	26-28.xi.1992	D Burckhardt	NHMB
fabianae	Chile	-29.9606	-70.1414	17.xii.1995	D Burckhardt	MHNG
fabianae	Chile	-29.9573	-70.0808	17.xii.1995	D Burckhardt	MHNG
fabianae	Chile	-37.8167	-73.0167	24-25.xii.1992	D Burckhardt	NHMB
fabianae	Chile	-38.2252	-71.8233	23-27.i.1996	D Burckhardt	MHNG
fabianae	Chile	-38.2252	-71.8233	23-27.i.1996	D Burckhardt	NHMB
fabianae	Chile	-36.8183	-71.7149	12.xii.1990	D Agosti & D Burckhardt	MHNG
fabianae	Argentina	-41.9549	-71.5325	4.xi.1961	G Topal	MHNG
fabianae	Argentina	-41.9549	-71.5325	4.xi.1961	G Topal	MHNG
fabianae	Argentina	-41.9678	-71.5155	4.xi.1961		BMNH
fabianae	Chile	-32.5061	-70.5650	6.iii.2009	D Burckhardt	NHMB
fabianae	Chile	-35.5500	-71.3333	4.i.1994	D Burckhardt	MHNG
fabianae	Chile	-35.6125	-71.0288	6.x.1983	LE Peña	MHNG
fabianae	Chile	-35.7500	-71.0000	25.xi.1992	D Burckhardt	NHMB
fabianae	Chile	-35.5833	-71.0667	4-5.i.1994	D Burckhardt	MHNG

fabianae	Chile	-35.5715	-70.9158	14.i.1996	D Burckhardt	MHNG
fabianae	Chile	-35.6275	-71.0723	13.i.1996	D Burckhardt	MHNG
fabianae	Chile	-35.6153	-71.0557	12.i.1996	D Burckhardt	MHNG
fabianae	Chile	-35.6000	-71.2000	25.xi.1993	D Burckhardt	NHMB
fabianae	Chile	-35.5852	-71.5679	14.ii.1985	D Hollis	BMNH
fabianae	Chile	-35.5954	-71.0515	14.ii.1985	D Hollis	BMNH
fabianae	Chile	-37.4228	-71.3645	19.i.1985	D Hollis	BMNH
intermedia	Bolivia	-20.5107	-65.1485	26–28.xii.1984	LE Peña	MHNG
intermedia	Bolivia	-20.5107	-65.1485	26–28.xii.1984	LE Peña	MHNG
intermedia	Bolivia	-20.1739	-65.2731	26/27.xii.1984	LE Peña	MHNG
lycii	Peru	-11.4116	-76.6137	27.ii.2014	D Whitmore	NHMB
lycii	Peru	-12.3649	-76.3714	16.x.1958	DL Tuthill	Burckhardt, 1987 [38]
maculata	Bolivia	-20.1739	-65.2731	26–27.xii.1984	LE Peña	MHNG
maculata	Argentina	-22.9760	-65.3525	31.xii.1984	LE Peña	MHNG
maculata	Bolivia	-20.1739	-65.2731	26/27.xii.1984	LE Peña	MHNG
marionae	Argentina	-32.5111	-69.0476	18.i.1997	D Burckhardt	NHMB
marionae	Argentina	-32.5111	-69.0476	18.i.1997	D Burckhardt	NHMB
nigra	Bolivia	-20.5107	-65.1485	26–28.xii.1984	LE Peña	MHNG
nigra	Bolivia	-20.5107	-65.1485	26–28.xii.1984	LE Peña	MHNG
nigra	Argentina	-32.5111	-69.0476	18.i.1997	D Burckhardt	MHNG
punctulata	Argentina	-22.9760	-65.3525	31.xii.1984	LE Peña	MHNG
punctulata	Argentina	-22.9760	-65.3525	31.xii.1984	LE Peña	MHNG
sebastiani	Chile	-37.3865	-71.3844	19.i.1996	D Burckhardt	MHNG
sebastiani	Chile	-35.5833	-71.0667	4-5.i.1994	D Burckhardt	MHNG
sebastiani	Chile	-35.5715	-70.9158	14.i.1996	D Burckhardt	MHNG
sebastiani	Chile	-35.6275	-71.0723	13.i.1996	D Burckhardt	MHNG
sebastiani	Chile	-35.6275	-71.0723	13.i.1996	D Burckhardt	NHMB
sebastiani	Chile	-35.6153	-71.0557	12.i.1996	D Burckhardt	MHNG
similis	Bolivia	-20.1739	-65.2731	26–27.xii.1984	LE Peña	MHNG
similis	Bolivia	-20.5107	-65.1485	26–28.xii.1984	LE Peña	NHMB
similis	Bolivia	-20.1739	-65.2731	26/27.xii.1984	LE Peña	MHNG
solanicola	Chile	-22.1041	-70.1065	23.iv.1993	D Burckhardt	MHNG
solanicola	Chile	-22.1041	-70.1065	23.iv.1993	D Burckhardt	MHNG
solanicola	Chile	-30.6095	-71.5443	19.ii.1985	D Hollis	BMNH
solanicola	Chile	-33.4718	-70.2954	19.xii.1950	ES Ross & AE Michelbacher	CAS
solanicola	Chile	-22.6404	-68.5881	23.iv.1993	D Burckhardt	MHNG
solanicola	Chile	-22.6404	-68.5881	23.iv.1993	D Burckhardt	MHNG
solanicola	Peru	-8.8671	-77.4244	22.v.3013	D Percy	NHMB
solanicola	Peru	-9.3333	-77.0516	26.v.2013	E McAlister	NHMB
solanicola	Brazil	-23.4158	-51.4264	30.vii.1996	C Wilcken	BMNH
solanicola	Argentina	-26.6795	-66.0482	21.iii.1974	CR Vardy	Burckhardt, 1987 [38]
solanicola	Argentina	-26.9589	-66.1464	26–28.xi.1983	LE Peña	Burckhardt, 1987 [38]
solanicola	Argentina	-40.8062	-62.9891	23.x.1926	F & M Edwards	Burckhardt, 1987 [38]
solanicola	Argentina	-24.7696	-65.4150	2–9.ii.1905	J Steinbach SJ	Burckhardt, 1987 [38]
solanicola	Argentina	-26.8224	-65.2213		Vezenyi	Burckhardt, 1987 [38]
solanicola	Chile	-27.3361	-70.7063	26.x.1983	LE Peña	MHNG
solanicola	Bolivia	-21.2574	-63.4586	xi.1930	Eisentraut	Burckhardt, 1987 [38]
solanicola	Brazil	-21.3687	-47.6848	20.vii.2006	M Rogers	FSCA
solanicola	Argentina	-26.6805	-66.0482	21.iii.1974	C R Vardy	BMNH

solanicola	Argentina	-28.4434	-65.7461	4.xi.1991	LE Peña	NHMB
solanicola	Chile	-26.3934	-70.0461	21.i.1992	LE Peña	NHMB
solanicola	Chile	-37.6713	-72.5875	1.iv.1932	DS Bullock	Burckhardt, 1987 [38]
solanicola	Chile	-33.0472	-71.6135		M Just sg	Burckhardt, 1987 [38]
solanicola	Chile	-32.8654	-71.4922	14.xii.1964	S Mahunka	Burckhardt, 1987 [38]
solanicola	Chile	-32.7847	-71.5349	27.x.1966	L Manne M	Burckhardt, 1987 [38]
solanicola	Bolivia	-20.5107	-65.1485	26–28.xii.1984	LE Peña	MHNG
solanicola	Bolivia	-20.5107	-65.1485	26–28.xii.1984	LE Peña	MHNG
solanicola	Chile	-27.8539	-70.5178	24.viii.1966	ME Irwin & EI Schlinger	CAS
solanicola	Chile	-27.8241	-70.7078	25.x.1991	LE Peña	NHMB
solanicola	Chile	-30.1673	-70.6627	17.xi.1987	LE Peña	MHNG
solanicola	Argentina	-31.3989	-64.1821	6.ii.1976		BMNH
solanicola	Peru	-13.5098	-71.9817	9.viii.1971	C Vardy	BMNH
solanicola	Peru	-13.3058	-72.1156	9.viii.1971	C Vardy	BMNH
solanicola	Argentina	-31.3980	-64.1821	6.ii.1976		MHNG
solanicola	Chile	-30.1673	-70.6627	23.x.1992	JG Rozen, A Sharkov, AJ Sayder	NHMB
solanicola	Chile	-30.0833	-70.6667	5.xii.1993	D Burckhardt	MHNG
solanicola	Chile	-29.8333	-70.0167	3.xii.1993	D Burckhardt	MHNG
solanicola	Chile	-29.5113	-71.2010	17.xi.1991	LE Peña	NHMB
solanicola	Chile	-30.2301	-70.4940	20.xii.1995	D Burckhardt	MHNG
solanicola	Peru	-9.9298	-76.2433	19-20.viii.1971	C Vardy	BMNH
solanicola	Chile	-28.8426	-70.8171	16.i.1992	LE Peña	NHMB
solanicola	Chile	-28.5761	-70.6033	22.ii.1985	D Hollis	BMNH
solanicola	Chile	-20.4773	-69.6764	24.i.1992	LE Peña	NHMB
solanicola	Chile	-30.5974	-71.5244	19.ii.1985	D Hollis	BMNH
solanicola	Chile	-32.9721	-71.0735	11.i.1985	D Hollis	BMNH
solanicola	Argentina	-29.4290	-66.8420	xi.1986	C Garcia	MHNG
solanicola	Chile	-29.9083	-71.2502	ii.1996	R Muniz	MHNG
solanicola	Peru	-10.7836	-76.8700	5.iii.2014	D Whitmore	NHMB
solanicola	Peru	-11.4974	-77.2095	12.ii.1995	C Vergara	NHMB
solanicola	Chile	-31.1078	-71.1997	01.xii.1950	ES Ross & AE Michelbacher	CAS
solanicola	Chile	-31.4295	-71.5078	13.xii.1950	ES Ross & AE Michelbacher	CAS
solanicola	Chile	-30.5974	-71.5244	19.ii.1985	D Hollis	MHNG
solanicola	Chile	-30.6557	-71.2584	12.xii.1950	ES Ross & AE Michelbacher	CAS
solanicola	Chile	-30.6667	-71.6667	15-17.xii.1992	D Burckhardt	NHMB
solanicola	Chile	-37.8082	-72.6982	21.xii.1924	DS Bullock	MHNG
solanicola	Chile	-37.8167	-73.0167	16-17.xii.1990	D Agosti & D Burckhardt	MHNG
solanicola	Chile	-37.8333	-73.0000	14-17.xii.1990	D Agosti & D Burckhardt	MHNG
solanicola	Chile	-37.8167	-73.0167	16-17.xii.1990	D Agosti & D Burckhardt	MHNG
solanicola	Chile	-37.8025	-73.0020	3.i.1951	ES Ross & AE Michelbacher	CAS
solanicola	Chile	-33.1667	-70.7833	15.xi.1992	D Burckhardt	NHMB
solanicola	Brazil	-21.2333	-45.0000	1-6.vi.2010	D Burckhardt	NHMB

solanicola	Brazil	-21.2333	-45.0000	1-6.vi.2010	D Burckhardt	NHMB
solanicola	Chile	-36.8695	-71.6163	12-13.xii.1990	D Agosti & D Burckhardt	MHNG
solanicola	Chile	-36.8695	-71.6163	12-13.xii.1990	D Agosti & D Burckhardt	MHNG
solanicola	Chile	-36.4322	-71.7601	24.xii.1950	ES Ross & AE Michelbacher	CAS
solanicola	Chile	-36.8183	-71.7149	12.xii.1990	D Agosti & D Burckhardt	MHNG
solanicola	Chile	-36.4356	-71.5320	24.xii.1950	ES Ross & AE Michelbacher	CAS
solanicola	Chile	-36.6116	-72.1026	4.i.1956	J Artigas	MHNG
solanicola	Chile	-36.6116	-72.1026	4.i.1956	J Artigas	NHMB
solanicola	Chile	-36.6116	-72.1026	4.i.1956	JN Artigas	MHNG
solanicola	Chile	-36.9202	-71.4931	12-13.xii.1983	LE Peña	MHNG
solanicola	Chile	-36.8866	-71.6280	12.xii.1991	D Agosti & D Burckhardt	MHNG
solanicola	Chile	-36.8808	-71.4890	13.xii.1990	D Agosti & D Burckhardt	MHNG
solanicola	Chile	-36.8808	-71.4890	13.xii.1990	D Agosti & D Burckhardt	NHMB
solanicola	Chile	-36.8808	-71.4890	13.xii.1990	D Agosti & D Burckhardt	MHNG
solanicola	Chile	-36.8808	-71.4890	13.xii.1990	D Agosti & D Burckhardt	MHNG
solanicola	Peru	-16.4078	-71.5373	19.xii.1957	JL Sánchez	Tuthill, 1959 [41]
solanicola	Peru	-16.4078	-71.5373	ii.1959		Tuthill, 1959 [41]
solanicola	Peru	-16.7328	-71.8721			Chávez et al. 2003 [92]
solanicola	Peru	-16.3520	-72.3092			Chávez et al. 2003 [92]
solanicola	Peru	-13.3022	-72.1142	9.vii.1971	C & M Vardy	Burckhardt, 1987 [38]
solanicola	Peru	-9.9340	-76.2387	19–20.viii.1971	C & M Vardy	Burckhardt, 1987 [38]
solanicola	Peru	-12.0523	-77.0480	xii.1957	JE Willie	Burckhardt, 1987 [38]
solanicola	Peru	-11.9014	-76.6638	28.x.1958	TD Tuthill	Burckhardt, 1987 [38]
solanicola	Peru	-17.1856	-70.9368			Chávez et al. 2003 [92]
solanicola	Peru	-18.0060	-70.2454			Chávez et al. 2003 [92]
solanicola	Chile	-32.5043	-71.3325	27.xi.1950	ES Ross & AE Michelbacher	CAS
solanicola	Chile	-32.4449	-71.3184	14.xii.1950	ES Ross & AE Michelbacher	CAS
solanicola	Chile	-32.5544	-71.4606	15.xii.1950	ES Ross & AE Michelbacher	CAS
solanicola	Chile	-32.8667	-71.2167	1.i.1994	D Burckhardt	MHNG
solanicola	Chile	-32.9721	-71.0735	11.i.1985	D Hollis	MHNG
solanicola	Chile	-32.9541	-71.1212	17.xii.1950	ES Ross & AE Michelbacher	CAS
solanicola	Bolivia	-20.1739	-65.2731	26/27.xii.1984	LE Peña	MHNG
solanicola	Chile	-32.5000	-70.7000	26.xii.1993	D Burckhardt	MHNG

solanicola	Chile	-32.6942	-70.7653	13–20.i.1997	D Burckhardt	MHNG
solanicola	Chile	-32.5619	-70.7048	24.xii.1995	D Burckhardt	MHNG
solanicola	Chile	-32.5619	-70.7048	24.xii.1995	D Burckhardt	MHNG
solanicola	Chile	-32.6844	-70.5946	27.xii.1998	D Burckhardt	NHMB
solanicola	Chile	-22.9162	-68.2023	24.iv.1993	D Burckhardt	MHNG
solanicola	Peru	-11.9019	-76.6658	28.x.1958	LD Tuthill	BMNH
solanicola	Brazil	-21.7248	-45.3569	2.ix.2014	D Burckhardt & DL Queiroz	NHMB
solanicola	Brazil	-21.7248	-45.3569	2.ix.2014	D Burckhardt & DL Queiroz	NHMB
solanicola	Bolivia	-15.6582	-69.1704	11.xi.1984	LE Peña	MHNG
solanicola	Chile	-35.1234	-71.6468	22.xii.1950	ES Ross & AE Michelbacher	CAS
solanicola	Chile	-35.5833	-71.0667	4-5.i.1994	D Burckhardt	MHNG
solanicola	Chile	-20.4891	-69.3290	23.ix–20.xi.1966	ME Irwin & E Medina	CAS
solanicola	Argentina	-26.8224	-65.2213	1986	C Garcia	MHNG
solanicola	Chile	-32.9721	-71.0735	11.i.1985	D Hollis	BMNH
solanicola	Chile	-32.8058	-71.4779	16.xii.1950	ES Ross & AE Michelbacher	CAS
solanicola	Chile	-32.8505	-71.4883	16.xii.1950	ES Ross & AE Michelbacher	CAS
sp	Chile	-28.5761	-70.6033	22.ii.1985	D Hollis	BMNH
sp	Chile	-28.5761	-70.6033	22.ii.1985	D Hollis	BMNH
sp	Chile	-30.1220	-70.4933	20.ii.1985	D Hollis	BMNH
sp	Chile	-30.1220	-70.4933	20.ii.1985	D Hollis	BMNH
sp	Argentina	-29.4128	-66.8560	1.xi.1986	C Garcia	BMNH
sp	Brazil	-21.2219	-43.7707	1.ii.1962	M Alvarenga	BMNH
sp	Peru	-12.5231	-76.5085	16.viii.1958	LD Tuthill	BMNH
sp	Peru	-12.5231	-76.5085	16.x.1958	LD Tuthill	BMNH
sp	Chile	-36.9032	-71.7640	16.i.1985	D Hollis	BMNH
sp	Chile	-36.9009	-71.6532	16.i.1985	D Hollis	BMNH
sp	Chile	-36.9009	-71.6532	16.i.1985	D Hollis	BMNH
theresae	Chile	-32.8333	-70.1333	1.xii.1993	D Burckhardt	MHNG
theresae	Chile	-32.9137	-70.0961	24.xii.1998	D Burckhardt	NHMB
theresae	Chile	-32.8660	-70.1481	23.xii.1995	D Burckhardt	MHNG
theresae	Chile	-32.8672	-70.1871	23.xii.1995	D Burckhardt	MHNG
theresae	Chile	-32.8920	-70.2276	23.xii.1995	D Burckhardt	MHNG
theresae	Chile	-32.8786	-70.2090	24.xii.1998	D Burckhardt	NHMB
theresae	Chile	-32.8333	-70.1333	31.xii.1993	D Burckhardt	MHNG
theresae	Chile	-32.8333	-70.1333	31.xii.1993	D Burckhardt	MHNG
theresae	Chile	-32.8333	-70.1333	23.xi.1992	D Burckhardt	MHNG
vinculipennis	Argentina	-22.9760	-65.3525	31.xii.1984	LE Peña	MHNG
vinculipennis	Chile	-30.2293	-70.4947	20.ii.1985	D Hollis	BMNH
vinculipennis	Chile	-30.1220	-70.4933	20.ii.1985	D Hollis	BMNH
vinculipennis	Chile	-30.1220	-70.4933	20.ii.1985	D Hollis	BMNH
vinculipennis	Bolivia	-20.1739	-65.2731	26/27.xii.1984	LE Peña	MHNG
vinculipennis	Chile	-29.5366	-71.2395	21.ii.1985	D Hollis	BMNH

S2 Table. Georeferenced *Russelliana* specimens. Specimen records in museum collections are as follows: Natural History Museum, London (BMNH), California Academy of Sciences (CAS), Museum d'Histoire Naturelle, Geneva (MHNG), Naturhistorisches Museum, Basel (NHMB) [38,41,92].
