# Supplementary material for: Emerging New Crop Pests: Ecological Modelling and Analysis of the South American Potato Psyllid Russelliana solanicola (Hemiptera: Psylloidea) and Its Wild Relatives
Source: PLoS One. 2017 Jan 4;12(1):e0167764. doi: 10.1371/journal.pone.0167764 (PMC5214844; doi:10.1371/journal.pone.0167764)
Supplement: S1 Fig — S1a Fig. Morphological traits. Boxplots of morphological traits for Russelliana species; Solanaceae feeding species (blue) and non-Solanaceae feeding species (orange). S1b Fig. Morphological traits. Boxplots of morphological traits for Russelliana species; Solanaceae feeding species (blue) and non-Solanaceae feeding species (orange). (PDF) [file pone.0167764.s003.pdf]

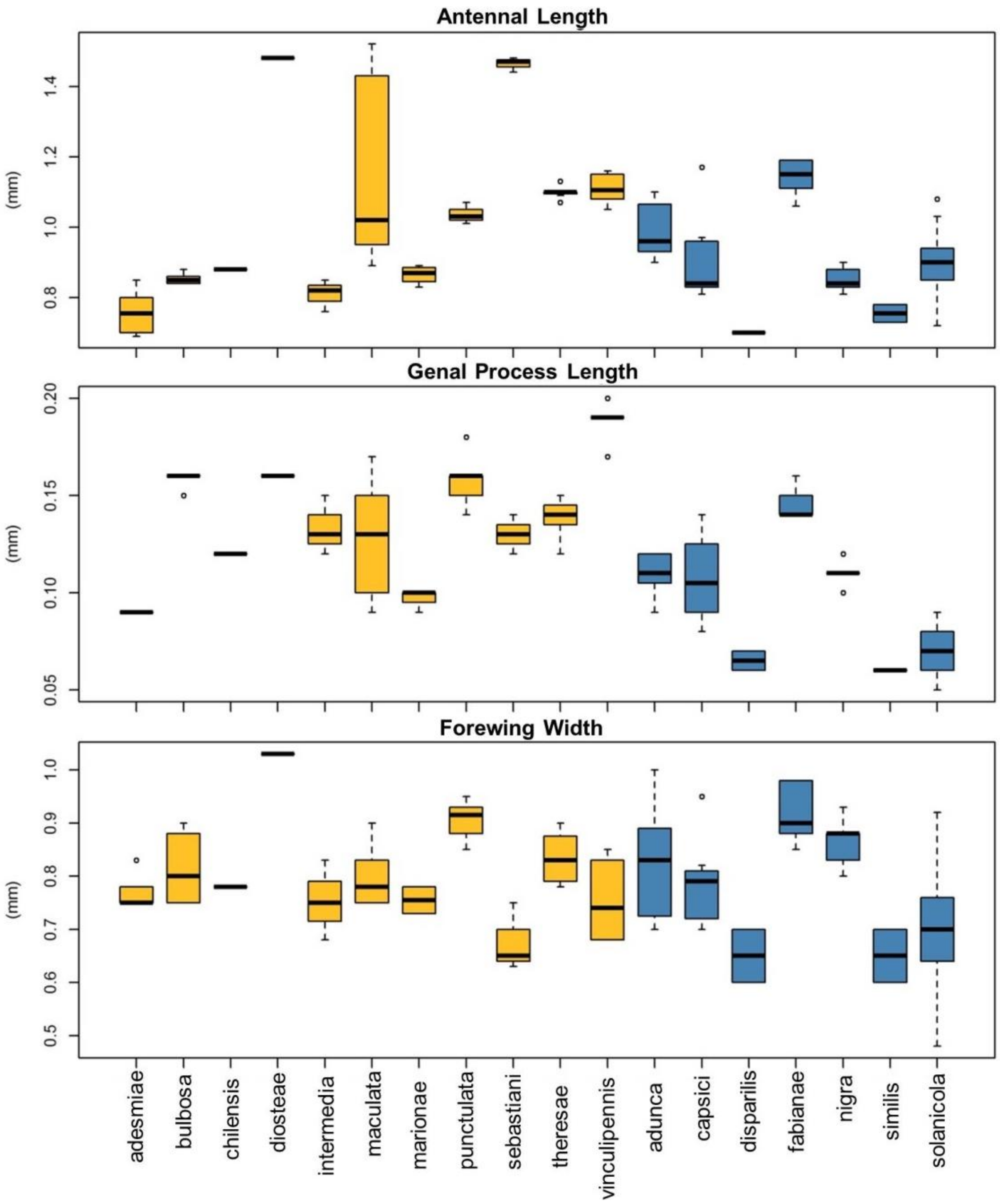

S1a Fig. Morphological traits. Boxplots of morphological traits for *Russelliana* species; Solanaceae feeding species (blue) and non-Solanaceae feeding species (orange).

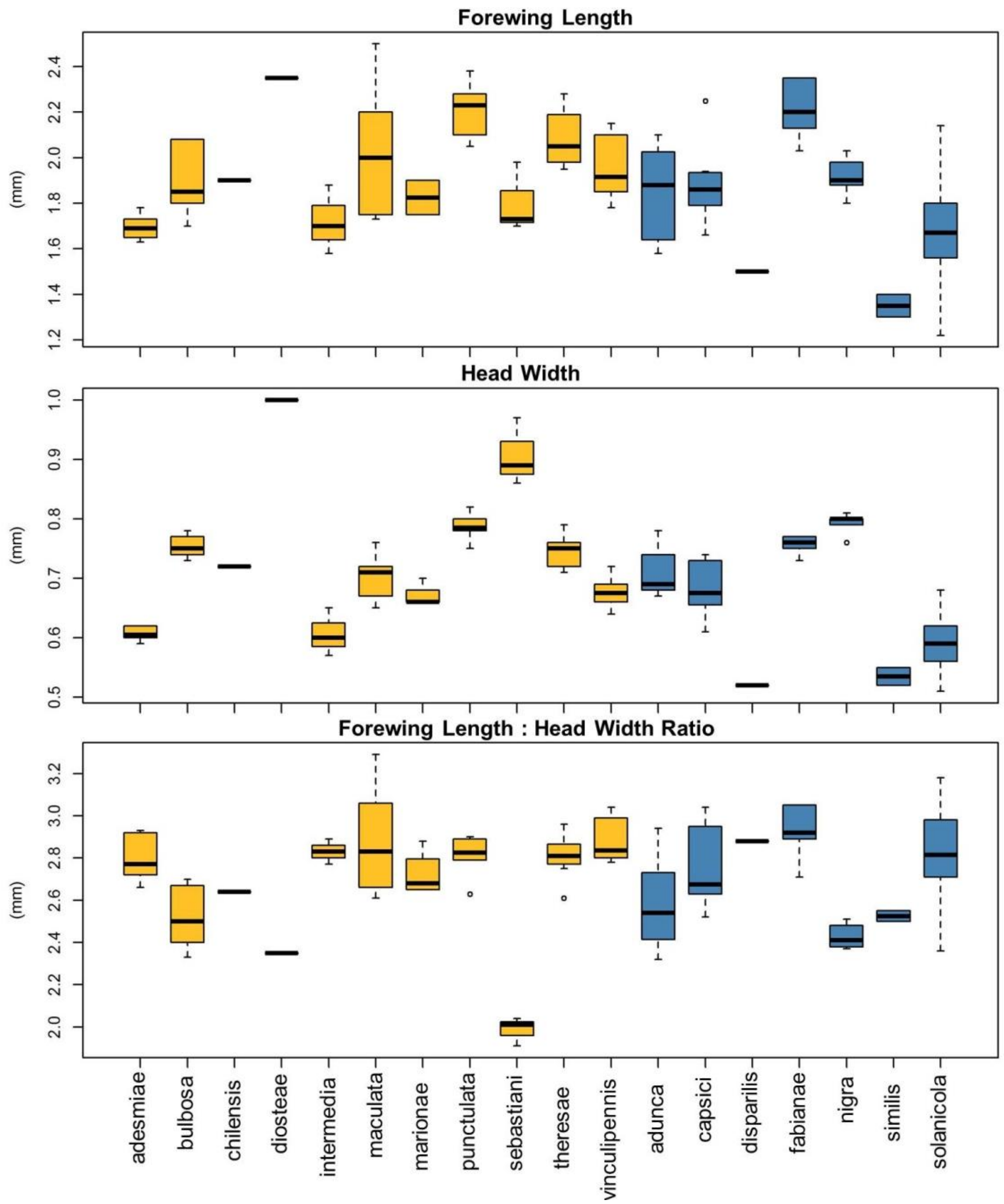

S1b Fig. Morphological traits. Boxplots of morphological traits for *Russelliana* species; Solanaceae feeding species (blue) and non-Solanaceae feeding species (orange).
