# Supplementary material for: Emerging New Crop Pests: Ecological Modelling and Analysis of the South American Potato Psyllid Russelliana solanicola (Hemiptera: Psylloidea) and Its Wild Relatives
Source: PLoS One. 2017 Jan 4;12(1):e0167764. doi: 10.1371/journal.pone.0167764 (PMC5214844; doi:10.1371/journal.pone.0167764)
Supplement: S2 Fig — Linear regressions of median values of morphological traits and geographical ranges for 19 Russelliana species. (PDF) [file pone.0167764.s004.pdf]

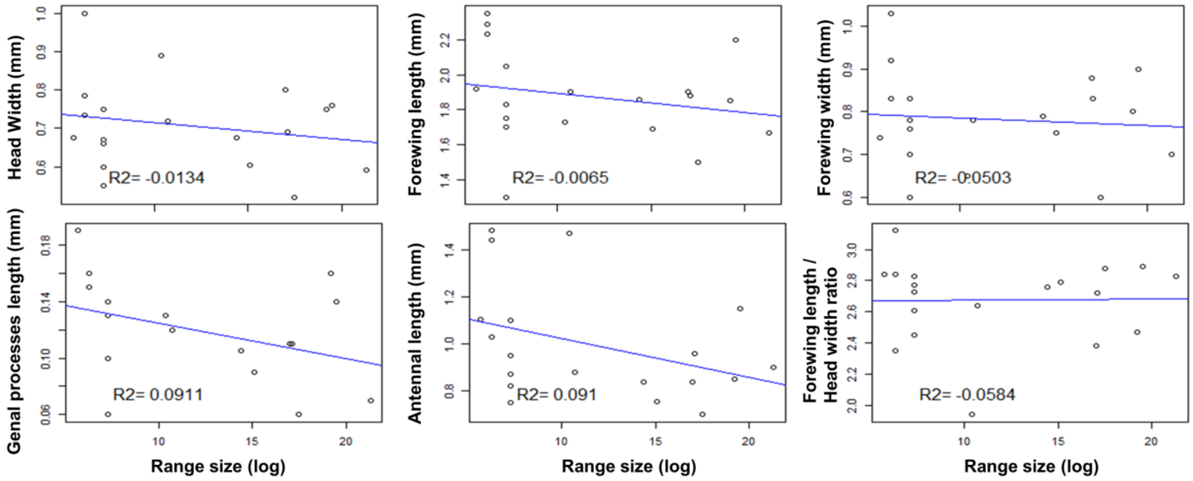

S2 Fig. Linear regressions morphological traits. Linear regressions of median values of morphological traits and geographical ranges for 19 *Russelliana* species.
