# Supplementary material for: Emerging New Crop Pests: Ecological Modelling and Analysis of the South American Potato Psyllid Russelliana solanicola (Hemiptera: Psylloidea) and Its Wild Relatives
Source: PLoS One. 2017 Jan 4;12(1):e0167764. doi: 10.1371/journal.pone.0167764 (PMC5214844; doi:10.1371/journal.pone.0167764)
Supplement: S3 Fig — Known geographical localities of 19 Russelliana species from Argentina, Bolivia, Brazil, Chile, Peru and Uruguay, overlapping with point data (grey) of potato crop wild relatives (CWR) available from Solanaceae Source (http://solanaceaesource.org/). Blue points represent Solanaceae feeding species and orange points represent non-Solanaceae feeding species. (PDF) [file pone.0167764.s005.pdf]

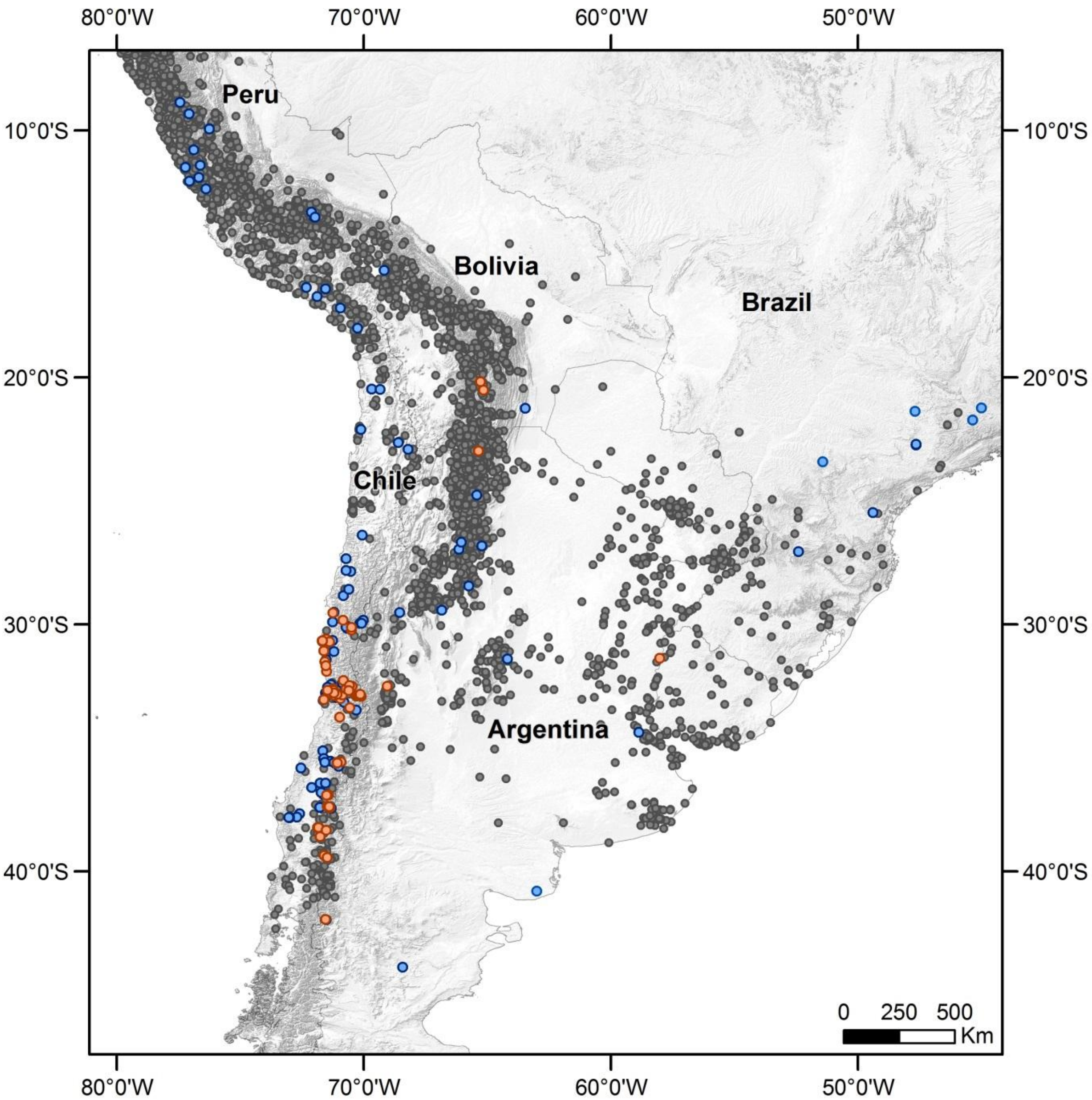

S3 Fig. Co-occurrence of *Russelliana* and potato wild relatives. Known geographical localities of 19 *Russelliana* species from Argentina, Bolivia, Brazil, Chile, Peru and Uruguay, overlapping with point data (grey) of potato crop wild relatives (CWR) available from Solanaceae Source (http://solanaceaesource.org/). Blue points represent Solanaceae feeding species and orange points represent non-Solanaceae feeding species.
